# Transport of Dietary Anti-Inflammatory Peptide, γ-Glutamyl Valine (γ-EV), across the Intestinal Caco-2 Monolayer

**DOI:** 10.3390/nu13051448

**Published:** 2021-04-24

**Authors:** Snigdha Guha, Sophie Alvarez, Kaustav Majumder

**Affiliations:** 1 Department of Food Science and Technology, University of Nebraska-Lincoln, Lincoln, NE 68588-6205, USA; snigdha.guha@huskers.unl.edu; 2 Proteomics and Metabolomics Facility, Nebraska Center for Biotechnology, University of Nebraska-Lincoln, Lincoln, NE 68588-0665, USA; salvarez@unl.edu

**Keywords:** peptide absorption, γ-glutamyl peptides, peptide transport mechanism, intestinal Caco-2 cells, bioactive peptides, P_app_

## Abstract

The present study analyzed the transepithelial transport of the dietary anti-inflammatory peptide, γ-glutamyl valine (γ-EV). γ-EV is naturally found in dry edible beans. Our previous study demonstrated the anti-inflammatory potency of γ-EV against vascular inflammation at a concentration of 1mM, and that it can transport with the apparent permeability coefficient (P_app_) of 1.56 × 10^−6^ ± 0.7 × 10^−6^ cm/s across the intestinal Caco-2 cells. The purpose of the current study was to explore whether the permeability of the peptide could be enhanced and to elucidate the mechanism of transport of γ-EV across Caco-2 cells. The initial results indicated that γ-EV was nontoxic to the Caco-2 cells up to 5 mM concentration and could be transported across the intestinal cells intact. During apical-to-basolateral transport, a higher peptide dose (5 mM) significantly (*p* < 0.01) enhanced the transport rate to 2.5 × 10^−6^ ± 0.6 × 10^−6^ cm/s. Cytochalasin-D disintegrated the tight-junction proteins of the Caco-2 monolayer and increased the P_app_ of γ-EV to 4.36 × 10^−6^ ± 0.16 × 10^−6^ cm/s (*p* < 0.001), while theaflavin 3′-gallate and Gly-Sar significantly decreased the P_app_ (*p* < 0.05), with wortmannin having no effects on the peptide transport, indicating that the transport route of γ-EV could be via both PepT1-mediated and paracellular.

## 1. Introduction

Dietary bioactive peptides have been shown to exhibit numerous health-modulating, beneficial biological activities, such as anti-inflammatory, antioxidant, antihypertensive, immunomodulatory, anticancer, and antithrombotic activities [1]. However, to exert such health-beneficial activities, these food-derived bioactive peptides must overcome two critical physiological barriers in order to be absorbed in the blood circulation intact. The first barrier is to survive the extensive hydrolysis in the gastrointestinal tract (GIT), and the second is to overcome the permeability across the intestinal epithelium [2]. A monolayer of human intestinal carcinoma-derived cell line, Caco-2, is generally used for transport and permeability studies of various drugs and peptides across the intestinal epithelium, as it mimics the human GIT in terms of the microvilli structure, tight junctions, and overall biological functions [3,4].

Bioactive peptides may range from 2 to 20 amino acid residues, and their transepithelial transport route depends upon their overall charge, molecular mass, hydrophobicity, and tendency to aggregate [5]. Numerous studies on the transport of food-derived bioactive peptides have been reported, and they may all have different transport mechanisms. The three main routes of peptide transport across the intestinal cells include: (i) the passive paracellular transport (via the tight junctions of the intestinal cells); (ii) transporter-mediated active transport, via the PepT1 transporter, particularly for di- and tripeptides; and (iii) transcytosis, which involves the endocytotic uptake of the peptide, vesicular transport within the cell, and basolateral secretion [6]. These routes of transepithelial transport were investigated using selective inhibitors or modulators for each of these specific routes. For instance, for the investigation of the paracellular pathway, transport was carried out in the presence of compounds such as cytochalasin D, which disrupted the tight junctions by modifying the cytoskeletal structure [7]. On the other hand, theaflavins enhanced the intestinal barrier by expressing more tight-junction proteins. Similarly, the compound glycyl-sarcosine (Gly-Sar) is a competitive inhibitor of the PepT1 transporter due to its higher affinity (lower K_m_ or Michaelis constant) for the transporter [8]. Finally, wortmannin is a specific noncompetitive and irreversible inhibitor of the phosphatidylinositol-3-kinase, and thus an inhibitor of transcytosis [9].

γ-glutamyl valine (γ-EV) is a naturally occurring bioactive dipeptide that is primarily found in foods such as legumes (i.e., common beans, soybeans, black gram), garlic, onions, cheese, and other fermented foods [10]. γ-EV has been characterized as an anti-inflammatory peptide, as it had previously been demonstrated to exhibit in vitro anti-inflammatory activities against vascular [11], gastrointestinal [12], and adipocyte [13] inflammation. The peptide had also shown in vivo anti-inflammatory effects in dextran sodium sulfate-induced mouse [12] and porcine models of colitis [14] and in lipopolysaccharide-induced mouse model of sepsis [15]. Despite these in vivo anti-inflammatory studies being present, none of them clearly acknowledged the mechanism of absorption of γ-EV across the intestinal layer. Our previous study evaluated the in vitro transport efficiency of the γ-EV peptide across the intestinal Caco-2 monolayer, and reported the apparent permeability (P_app_) of γ-EV to be 1.56 × 10^−6^ cm/s [11]. The P_app_ value of γ-EV was found comparable to other dietary bioactive peptides, which were evaluated in both in vitro and in vivo studies [5,16,17,18]. Thus, the present study investigates how the current P_app_ of γ-EV could be improved and elucidates the mechanisms of transport of γ-EV across the Caco-2 monolayer as a useful model to explore the peptide transport.

## 2. Materials and Methods

### 2.1. Chemicals

The γ-EV peptide was synthesized chemically (purity >98%) through GenScript Inc., Piscataway, NJ, USA. All the chemicals used for the transport mechanism study were obtained from Millipore Sigma, Burlington, MA, USA: theaflavin 3′-gallate (#PHL83343), wortmannin (#W1628), cytochalasin D (#C8273), Gly-Sar (#G3127), 4′,6-Diamidino-2-phenylindole (DAPI) (#D9542), paraformaldehyde (#158127), Triton X-100 (#T8787), bovine serum albumin (BSA) (A2153), 4-(2-Hydroxyethyl) piperazine-1-ethanesulfonic acid (HEPES) (H4034), and 4-Morpholineethanesulfonic acid (MES) (M3671). The primary antibodies used in immunofluorescence (IF) and Western immunoblotting (WB) were as follows: anti-zonula occludens-1 (ZO-1, 1:20 (IF) and 1:200 (WB) dilutions) (Invitrogen, #61-7300), anti-claudin-1 (Invitrogen, #37-4900, 1:50 (IF) and 1:100 (WB) dilutions), anti-occludin (Invitrogen, #33-1500, 1:50 (IF) and 1:200 (WB) dilutions), and anti-β-actin (Sigma, #A5316, 1:5000 dilution). The secondary antibodies used for the Western immunoblotting experiments were IRDye 800RD goat anti-rabbit antibody (Li-Cor, #926-32211, 1:10,000 dilution) and IRDye 680RD goat anti-mouse antibody (Li-Cor, #926-68070, 1:10,000 dilution). The secondary antibodies used for the immunofluorescence experiments were goat anti-rabbit (Alexa Fluor 488) (Abcam, #ab150077, 1:500 dilution) and goat anti-mouse (Alexa Fluor 594) (Abcam, #ab150116, 1:500 dilution).

### 2.2. Caco-2 Cell Culture

Caco-2 (ATCC^®^ HTB-37™) cells, at a passage number between 24–26, were used for the experiments. Eagle’s Minimum Essential Medium (EMEM) (ATCC^®^ 30-200) with 20% fetal bovine serum (FBS) (Gibco, #10-437-028) and 1% penicillin-streptomycin (Pen-Strep) (10,000 U/mL) (Gibco, #15140122) was used for the proliferation of Caco-2 cells. The cells were kept in a humidified environment at 37 °C and 5% CO_2_. Caco-2 cells were proliferated until they were 80% confluent, with media being replaced with fresh media every other day, followed by sub-culturing onto 12 mm Transwell inserts with 0.4 μm pore polyester membrane (Corning, #3460) (50,000 cells/insert) for the transport experiments.

### 2.3. Cell Viability Assay

A clear-bottom 96-well black plate (VWR, #29444-008) was seeded with Caco-2 cells (40,000 cells/well) and grown for 15 days in EMEM media (20% FBS and 1% Pen-Strep) to achieve differentiation of the cells. The MTT assay kit (Abcam, #ab211091) was used to test the cell-viability assay following the manufacturer’s guidelines, as described in the earlier study [11]. For the time-course study, a concentration of 1 mM γ-EV was used for determining the cell toxicity, by incubating it on the Caco-2 cells for 2, 4, and 6 h. For the dose study, the cell toxicity of γ-EV was tested for different doses, such as 2.5, 3, 4, 5, and 10 mM, after incubating the peptide on the cells for 2 h. After the treatment period, the cell media was discarded and replaced with a mixture of 50 μL of the MTT reagent and 50 μL of serum-free EMEM media. After the incubation period of 3 h at 37 °C, the MTT-supplemented media was replaced with 150 μL of MTT solvent. Following that, the plates were incubated on an orbital shaker (200 rpm, 37 °C) (MaxQ 4450, Thermo Fischer Scientific, Waltham, MA, USA) for 15 min, in dark, after which the absorbance of the plate was read at 590 nm (Synergy H1 microplate reader, BioTek, Winooski, VT, USA). The background control readings were subtracted from each of the wells and the cell-viability percentage (%) was calculated using the formula cell viability % = (sample/control) × 100, where the control was cells with no γ-EV treatment, and the sample was the γ-EV treated cells.

### 2.4. Transport Assay

In order to determine whether the current P_app_ of γ-EV could be improved, two sets of transport experiments were performed: first, with varying the time of γ-EV incubation; and second, with varying the dose of γ-EV. The transport studies were performed as described in an earlier study [11]. Briefly, for both experiments, Caco-2 cells were grown onto the 12-well Transwell inserts (50,000 cells/insert) for 21 days, and the media was changed every other day. The apical side contained 0.5 mL of media, while the basal side of the inserts contained 1.5 mL of media. The transepithelial electrical resistance (TEER) values of the inserts were measured on the alternate days continuously for 21 days using EVOM2 paired with STX2 chopstick electrodes (World Precision Instruments, Sarasota, FL, USA). Caco-2 cells containing wells with EMEM growth medium with a TEER value greater than or equal to 400 Ωcm^2^ were selected for the transport experiments. The TEER values greater than 260 ± 65 Ωcm^2^ are generally recommended for the transport study [4]. On the 21st day, the EMEM media was removed and the warm Hank’s Balanced Salt Solution (HBSS) (Gibco, #14025076) was used to wash the cells. The apical layer was then replaced with HBSS at a pH of 6.5 (pH adjusted with 10 mM MES), while the basal layer was replaced with HBSS at a pH of 7.4 (pH adjusted with 25 mM HEPES). The cells were kept for 15 min at 37 °C and 5% CO_2_, after which the TEER values were measured again before the start of the experiment.

For the time-course study, 1 mM γ-EV dissolved in 0.5 mL of HBSS (pH 6.5) was replaced in the apical layer of the inserts with the basolateral layer still containing 1.5 mL HBSS at pH 7.4. The plate was incubated at 37 °C (MaxQ 4450, Thermo Fischer Scientific, Bedford, MA, USA) on an orbital shaker (200 rpm) for 2, 4, and 6 h. At the end of each time point, the apical and basal layer solutions were collected, and the amount of γ-EV transported was analyzed via liquid chromatography coupled with tandem mass spectrometry (LC-MS/MS). A similar approach was used for the variable dose study, where instead of using 1 mM, two higher doses of γ-EV (2.5 mM and 5 mM) were dissolved in 0.5 mL HBSS (pH 6.5) and replaced in the apical layer of the inserts. In this case, the plate was incubated at 37 °C for 2 h on an orbital shaker, after which the apical and basal layer solutions were collected and analyzed by LC-MS/MS. The P_app_ of the peptide was calculated by the following formula: P_app_ = (d*Q*/d*t*) (1/AC_0_), where d*Q*/d*t* is the transport rate (*μ*mol/Ls), A is the surface area of the inserts, and C_0_ is the initial concentration of the peptide concentration on the apical side (*μ*mol/L).

In all the cases, after collecting the solutions, the Caco-2 cells were also harvested from the inserts using 80 μL of RIPA lysis and extraction buffer (Thermo Scientific, #89900) for the analysis of the tight-junction proteins through Western immunoblotting.

### 2.5. Transport-Mechanism Assay

The route of transport of γ-EV across Caco-2 monolayer cells was determined using 1 mM γ-EV transported for 2 h, following the same protocol as mentioned in Section 2.4. However, in this case, four transport inhibitors were used prior to γ-EV addition in the apical layer, to elucidate the mechanism of transport. Theaflavin 3′-gallate (TF3′G) (20 μM), a cell tight-junction enhancer, was added to the EMEM growth medium 24 h before the experiment. Wortmannin (a transcytosis inhibitor, 1 μM), cytochalasin D (a cell tight-junction disruptor, 0.5 μg/mL), and Gly-Sar (PepT1 transporter inhibitor, 25 mM) were added 30 min before γ-EV addition in the HBSS buffer (pH 6.5). After the treatment was over, the apical and basal layers were collected, and the concentration of γ-EV was analyzed and represented via P_app_. For this study, some of the replicate inserts were used for harvesting the Caco-2 cells for analyzing the tight-junction proteins via Western immunoblotting, while some of the inserts were used for visualizing the tight-junction proteins through immunofluorescence.

### 2.6. Western Immunoblotting

The Caco-2 cell lysates were mixed with 2×Laemmli buffer to load 30 μg protein into each well of a 4−20% gradient gel (Biorad, #4561096), and Western immunoblotting was performed as described earlier [11]. The proteins were separated by sodium dodecyl sulfate−polyacrylamide gel electrophoresis (SDS-PAGE), after which they were transferred onto a nitrocellulose membrane (Bio-Rad, Hercules, CA, USA). After the transfer, the membranes were blocked for 1.5 h using the Odyssey blocking buffer in TBS (Li-Cor, #927-50000), after which they were incubated overnight at 4 °C with the desired antibodies and diluted in Odyssey blocking buffer at their recommended dilutions (as mentioned in Section 2.1). The next day, the membranes were washed (x3) with Tris-Buffered Saline, 0.1% Tween 20 (TBST) and incubated with the secondary antibodies (IRDye 680RD goat anti-mouse IgG and/or IRDye 800CW goat antirabbit IgG) at room temperature for 1 h in dark. The membranes were again washed (x2) with TBTS, and the proteins bands were detected via red and green fluorescent channels through the Odyssey CLx imaging system (Li-Cor Biosciences). Densitometrical quantification was done for the protein expression using the Image Studio software from Li-Cor, and β-actin expression was used as a loading control to normalize each blot.

### 2.7. Immunofluorescence

Once apical layer solutions were collected, the Caco-2 monolayer cells on the inserts were washed immediately with warm phosphate buffered saline (PBS), and cells were not allowed to dry from this point on. For fixation, 250 μL of 4% paraformaldehyde (PFA) (dissolved in warm PBS) was added onto the cells for 20 min at room temperature. The PFA was then discarded, and the cells were washed gently with PBS, 3 times for 5 min each, on a rocker. Following that, 250 μL of permeabilizing buffer (0.1% Triton X-100 in PBS) was added to each insert for 5 min at room temperature. After incubation, the buffer was removed and the cells were again washed with PBS, 3 times for 5 min each. Next, 250 μL of blocking buffer (1% BSA in PBS) was added to each well and kept for 60 min at room temperature on a rocker. After blocking, the buffer was removed and washed 3 times with PBS, and then 250 μL of the desired primary antibody solution (in 0.1% BSA in PBS) was added to each well. The plate was sealed and stored at 4 °C overnight. The next day, the primary antibody solution was removed, and the cells were washed 3 times with PBS, after which 250 μL of the respective secondary antibody solution (1:500 dissolved in 1% BSA in PBS) was added to the inserts for 60 min at room temperature and in dark conditions. After 60 min, the secondary antibody was removed and the cells were washed with PBS 3 times, and then 250 μL of the nuclear stain DAPI (1:10,000 in 0.1% BSA in PBS) was added to the inserts for exactly 10 min. After DAPI incubation was over, the cells were washed with PBS 3 times and the plate was covered to protect it from light, and the images were captured via EVOS Cell Imaging Systems (Thermo Fischer Scientific, Bedford, MA, USA).

### 2.8. Liquid Chromatography-Mass Spectrometry/Mass Spectrometry (LC-MS/MS)

LC-MS/MS was used to detect the γ-EV content after the transport study. The instrument used was the Shimadzu LC system (Nexera X2, Columbia, MD, USA), interfaced with a Sciex QTRAP 6500+ mass spectrometer (Redwood City, CA, USA), equipped with a TurboIonSpray (TIS) electrospray ion source (ESI). The LC separation was achieved with an Agilent Eclipse XDB-C18 (3.5 μm, 100 × 3.0 mm, Santa Clara, CA, USA) at a flow rate of 0.5 mL/min. The mobile phases and gradient used were as previously described [11]. Analyst software (version 1.6.3, Redwood City, CA, USA) was used for the sample acquisition control and data analysis. The manufacturer’s recommendations were used for tuning and calibration of the SCIEX QTRAP 6500+ mass spectrometer. The parameters for the ESI operation were as follows: 5500 V ion spray voltage; 500 °C source temperature; ion source gas 1, gas 2, and curtain gas, as 50, 50, and 25, respectively; and collision gas, high. The multiple reaction monitoring (MRM) transitions and the compound settings were previously optimized and described in [11]. An external standard curve, containing different concentrations of the peptide (γ-EV diluted in water), was prepared for the absolute quantification of the peptide. The standards were run alongside the samples (γ-EV in HBSS buffer) in triplicate. The linear range of the standard curve was from 0.016 to 10 μM, with a linear regression slope R^2^ of 1 with a CV of <8% for all the dilution points.

### 2.9. Statistical Analysis

Multiple groups were analyzed using Tukey’s multiple comparison test, following a one-way analysis of variance (ANOVA) using GraphPad Prism software (version 8.0.2, San Diego, CA, USA). Before the ANOVA analysis, each data set was analyzed via the D’Agostino–Pearson test to confirm the normal distribution. Data were presented as ± standard deviation (SD) of the mean, and differences were considered statistically significant with *p* < 0.05.

## 3. Results

### 3.1. Cytotoxicity in Caco-2 Cells

**Figure 1:**
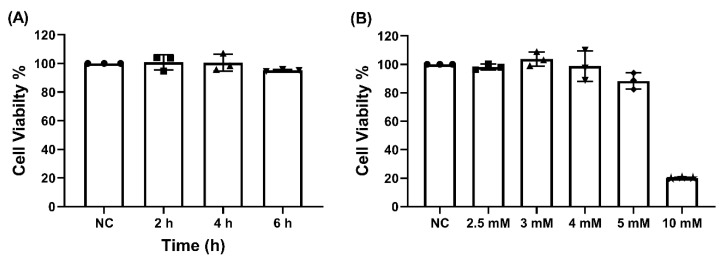
The cytotoxicity of γ-EV on Caco-2 cells. (**A**) No cytotoxicity was observed for 1 mM γ-EV when incubated on Caco-2 cells for elongated times of 4 and 6 h. NC: Negative control, did not receive γ-EV. (**B**) γ-EV was not cytotoxic to Caco-2 cells up to 5 mM concentration, while a 10 mM concentration was cytotoxic to the cells. Data are presented as the mean ± SD from n = 3 independent experiments.

The MTT cell viability assay demonstrated that γ-EV at a concentration of 1 mM was not toxic to the Caco-2 cells, even after prolonged exposures of 4 and 6 h (Figure 1A). Similarly, it was found that γ-EV, at concentrations up to 5 mM, was not toxic to the Caco-2 cells when exposed for 2 h. However, at a 10 mM concentration, the survival rate dropped to 21% after 2 h (Figure 1B). Therefore, for the variable-dose study, the optimal concentrations of 2.5 and 5 mM were used.

### 3.2. Transepithelial Transport of γ-EV across Caco-2 Monolayer Cells

**Figure 2:**
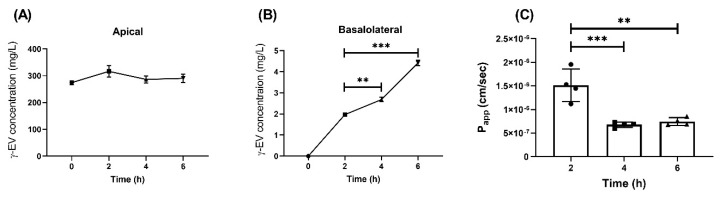
Time-dependent transport of 1 mM γ-EV across Caco-2 cells. (**A**) Concentration of γ-EV at different time points (2, 4, and 6 h) in the apical compartment. (**B**) Time-dependent increase in the concentration of γ-EV in the basolateral compartment after 4 and 6 h. (**C**) The rate of transport (P_app_) was found to be the highest up to 2 h, after which it significantly reduced at 4 and 6 h. Data are presented as the mean ± SD from n = 4 independent experiments; *, **, and *** indicate *p* < 0.05, *p* < 0.01, and *p* < 0.001, respectively.

To determine whether the current P_app_ of 1 mM γ-EV, i.e., 1.56 × 10^−6^ ± 0.7 × 10^−6^ cm/s (reported in our previous study [11]), could be improved or not, a prolonged exposure of 1 mM γ-EV was used for the transepithelial transport across Caco-2 monolayer cells. As depicted in Figure 2A, the peptide remained stable and intact in the apical layer for up to 6 h (Figure S1A,B). It was found that the total concentration of the γ-EV peptide significantly increased in the basolateral region over periods of 4 (*p* < 0.01) and 6 h (*p* < 0.001) as compared to 2 h (Figure 2B); however, the calculated P_app_ values for each of the time points showed that 2 h (1.56 × 10^−6^ ± 0.7 × 10^−6^ cm/s) had a significantly higher P_app_ value (*p* < 0.01) as compared to 4 h (6.78 × 10^−7^ ± 0.56 × 10^−7^ cm/s) and 6 h (7.46 × 10^−7^ ± 0.8 × 10^−7^ cm/s) (Figure 2C). This was probably due to the fact that although the peptide concentration was increasing over time and 6 h had the highest concentration of γ-EV, the rate of transport was the highest until 2 h, after which the rate began decreasing as the time progressed. Furthermore, the expression levels of the tight-junction proteins ZO-1, occludin, and claudin-1 were analyzed after the prolonged transport, and no significant differences were found among the expression levels of these proteins across the different time points, suggesting that the tight-junction barrier and stability were not altered due to the transport (Figure S2).

**Figure 3:**
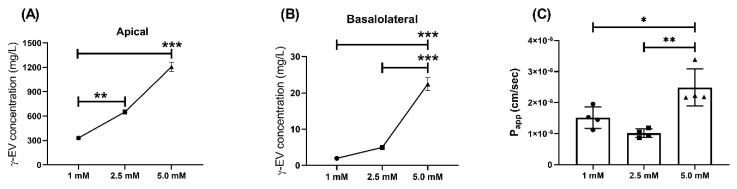
Dose-dependent transport of γ-EV across Caco-2 cells for 2 h. (**A**) Concentration of different doses of γ-EV (1, 2.5, and 5 mM) in the apical compartment after 2 h. (**B**) Dose-dependent increase in the concentration of γ-EV in the basolateral compartment after 2 h. (**C**) Dose-dependent increase in the rate of transport (P_app_) of 5 mM γ-EV as compared to 1 and 2.5 mM. Data are presented as the mean ± SD from n=4 independent experiments; *, **, and *** indicate *p* < 0.05, *p* < 0.01, and *p* < 0.001, respectively.

Similarly, the effect of higher doses, i.e., 2.5 and 5 mM, on the P_app_ of γ-EV was analyzed over a period of 2 h. For 2.5 and 5 mM doses, the concentration of the peptide was significantly higher than for the 1 mM dose in both apical and basal layers after 2 h (Figure 3A,B). Furthermore, it was also found that the P_app_ value of 5 mM (2.49 × 10^−6^ ± 0.6 × 10^−6^ cm/s) was significantly increased (*p* < 0.01) as compared to the 1 mM dose, while 2.5 mM did not have a significant change in the rate of transport (Figure 3C). This suggested that the flux rate was concentration dependent. Furthermore, analysis of the tight-junction proteins revealed that ZO-1 and claudin expressions were not affected by the increase in dosage; however, in case of occludin, there was a significant decrease (*p* < 0.05) in expression for the 5 mM dosage as compared to 1 mM, suggesting that the higher peptide dose might modulate the tight-junction proteins (Figure S3).

### 3.3. Mechanism of γ-EV Transport across the Caco-2 Monolayer

**Figure 4:**
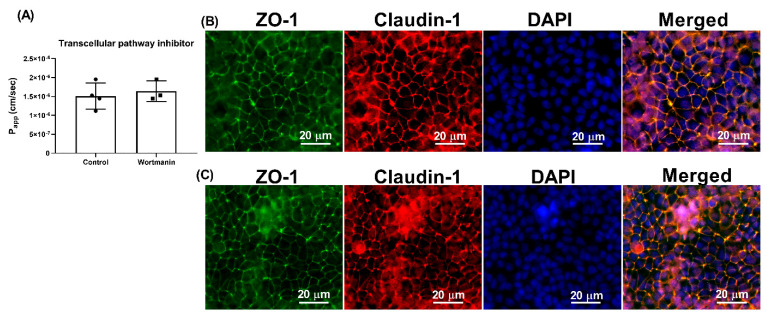
Effect of wortmannin on the transport of γ-EV across Caco-2 monolayer cells. (**A**) The transport of 1 mM γ-EV assessed over a period of 2 h, after the pretreatment of wortmannin (1 μM, 30 min). (**B**) Immunofluorescence staining of the tight-junction proteins zona occludin-1 (ZO-1) and claudin-1 of the wortmannin-treated Caco-2 monolayer cells on the membrane, to ensure the maintenance of cell integrity after 2 h of transport. 4′,6-Diamidino-2-phenylindole (DAPI) was used to stain the nucleus of the cells. (**C**) Immunofluorescence staining of the tight-junction proteins ZO-1 and claudin-1 on the Caco-2 monolayer cells on the membrane, without any treatment (control), to ensure the maintenance of cell integrity after 2 h of transport. Data presented as the mean ± SD from n = 3 (wortmannin) and n = 4 (control) independent experiments.

To investigate the route of transepithelial transport of γ-EV across Caco-2 cells, several inhibitors were used, such as wortmannin, TF3′G, cytochalasin D, and Gly-Sar. As seen in Figure 4A, the addition of 1 μM wortmannin had no significant difference in the P_app_ of γ-EV as compared to the control (without any inhibitor), suggesting that the transport was not through the transcytosis pathway.

**Figure 5:**
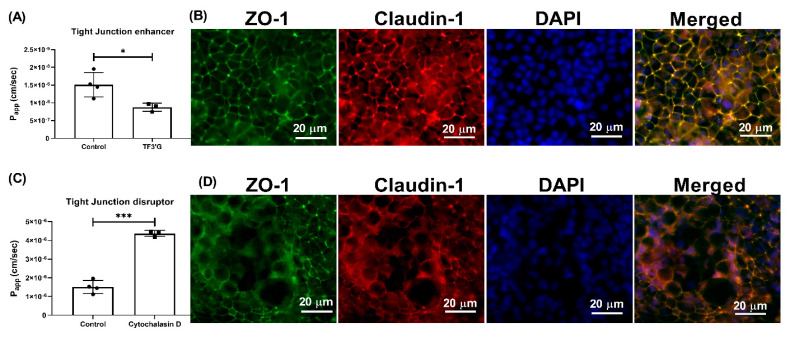
Effect of TF3′G and cytochalasin D on the transport of γ-EV across Caco-2 monolayer cells. (**A**) The transport of 1 mM γ-EV, assessed over a period of 2 h, after the pretreatment of TF3′G (20 μM, 24 h). (**B**) Immunofluorescence staining of the tight-junction proteins ZO-1 and claudin-1 of the TF3′G-treated Caco-2 monolayer cells on the membrane, to ensure the maintenance of cell integrity after 2 h of transport. (**C**) The transport of 1 mM γ-EV, assessed over a period of 2 h, after the pretreatment of cytochalasin D (0.5 μg/mL, 30 min). (**D**) Immunofluorescence staining of the tight-junction proteins ZO-1 and claudin-1 of the cytochalasin-treated Caco-2 monolayer cells on the membrane, to ensure the maintenance of cell integrity after 2 h of transport. Control immunofluorescence images are same as those mentioned in Figure 4. Data are presented as the mean ± SD from at least n = 3 (TF3′G and cytochalasin D) and n = 4 (control) independent experiments; *, and *** indicate *p* < 0.05 and *p* < 0.001, respectively.

Pretreatment of the Caco-2 cells with TF3′G (20 μM), a tight-junction enhancer, significantly decreased (*p* < 0.05) the P_app_ of γ-EV, to 8.75 × 10^−7^ ± 1.15 × 10^−7^ cm/s, when compared to the control (Figure 5A), suggesting that a passive paracellular route via tight junctions might be involved (Figure 5B). With the pretreatment of 20 μM TF3′G for 24 h, we observed a 44% reduction in γ-EV transport, which was in accordance with other studies reported in the literature using the same dose, such as the peptides LKP (reduced by 44%) and IQW (reduced by 45%) [19]. Furthermore, use of cytochalasin D (0.5 μg/mL), a tight-junction disrupter, significantly increased (*p* < 0.001) the transport of γ-EV by 64% (Figure 5C), which reinstated the role of tight junctions in the mechanism of transport (Figure 5D). The dose used was again in accordance with the literature; for instance, in the cases of the peptides HLPLP [20], VGPV [21], and GPRGF [21], pretreatment of 0.5 μg/mL cytochalasin D also increased the peptide transport by 65% across Caco-2 cells.

**Figure 6:**
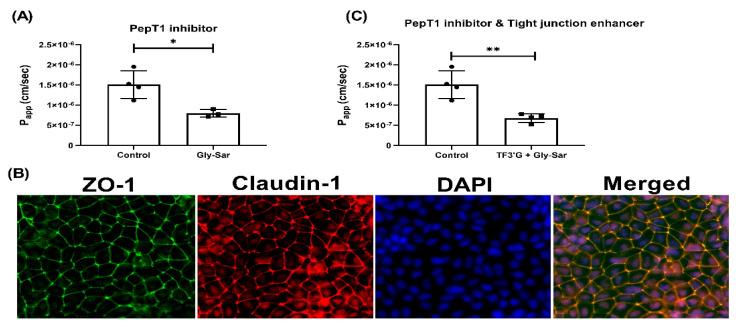
Effect of Gly-Sar on the transport of γ-EV across Caco-2 monolayer cells. (**A**) The transport of 1 mM γ-EV, assessed over a period of 2 h, after the pretreatment of Gly-Sar (25 mM, 30 min). (**B**) Immunofluorescence staining of the tight-junction proteins ZO-1 and claudin-1 of the Gly-Sar-treated Caco-2 monolayer cells on the membrane, to ensure the maintenance of cell integrity after 2 h of transport. (**C**) The transport of 1 mM γ-EV, assessed over a period of 2 h, after the pre-treatment of both TF3’G and Gly-Sar simultaneously. Control immunofluorescence images are same as those mentioned in Figure 4. Data presented as the mean ± SD from at least n = 3 (Gly-Sar) and n = 4 (control and TF3′G/Gly-Sar) independent experiments; * and ** indicate *p* < 0.05 and *p* < 0.01, respectively.

In addition, it was also found that pretreatment with Gly-Sar (25 mM), which is a specific PepT1 inhibitor, also significantly decreased the P_app_ of γ-EV to 7.98 × 10^−7^ ± 0.93 × 10^−7^ cm/s (*p* < 0.05), or by 48% as compared to the control, suggesting that the transport was also PepT1-mediated (Figure 6A). Similar results were also found in the literature for other bioactive peptides, such as the egg-derived antihypertensive peptide IRW, for which pretreatment of 25 mM of Gly-Sar reduced the transport by 44% [22]. Additionally, to confirm the two routes of transport, i.e., paracellular and PepT1, pretreatment of Caco-2 cells with both TF3′G and Gly-Sar together, at their respective concentrations, showed that the transport was further reduced by 56.41% (Figure 6C). However, the γ-EV transport was not completely abolished, suggesting that the PepT1 inhibitor was probably not 100% effective, and that using a PepT1 knock-out cell model could be more effective. Additionally, the tight-junction enhancer also had its own limitations, and thus the peptide transport was not completely attenuated. Furthermore, no significant differences were found in the tight-junction proteins among different treatment groups, suggesting that the use of chemical inhibitors did not interfere with the tight-junction barrier or stability (Figure S4). In sum, it was found that γ-EV was able to transport across the Caco-2 monolayer via two pathways, i.e., via the paracellular pathway, as well as through the PepT1 transporter.

## 4. Discussion

The current study is a follow-up study of our previous work, which established the efficacy of γ-EV for its anti-inflammatory activity against vascular inflammation at 1 mM dosage, and also investigated the P_app_ of the peptide for the same dose [11]. The present study, however, aimed to analyze methods to increase the P_app_ of the peptide, as well as to elucidate the mechanism of transport of the peptide across the intestinal cells. Our study showed that 1 mM γ-EV can transport across the Caco-2 cells over a prolonged period of time, i.e., up to 6 h, without becoming degraded. After 4 and 6 h of transport, the basolateral layers had intact γ-EV, without any fragmentation (Figure S5A,B), which is in accordance with the fact that γ-glutamyl peptides are generally resistant to degradation, for instance by the brush border peptidases, due to the presence of the unusual γ-bond. This is an important factor to consider, as γ-EV must cross the intestinal epithelium intact to exert its anti-inflammatory bioactivity on various cells. Additionally, it was also observed that despite a continuous transport of the peptide across the monolayer cells for 6 h, the rate of transport was the maximum in the initial 2 h, after which the transport slowed down, probably because of saturation of the transporter protein [5]. Furthermore, the peptide transport was found to be concentration-dependent because a higher γ-EV dose of 5 mM significantly increased the rate of transport. This phenomenon, along with a slight decrease in the occludin tight-junction protein (*p* < 0.05) for the 5 mM dose (Figure S3B), suggested the fact that the transport also was possibly paracellular as well, as it involved the tight-junction proteins.

In general, there are three main transport routes across Caco-2 cells: transcytosis, paracellular, and transporter-mediated. Wortmannin, an inhibitor of phosphoinositide 3-kinase, inhibits the endocytosis pathway. Since use of wortmannin did not alter the transport of γ-EV (Figure 4), this suggested it was not the route of transport. Studies have reported that transcytosis is actually tended by larger peptides, such as bradykinin [17], cationic peptide [23], β-casein [17], and YPFPG [6]. While transcytosis pathway is mainly used by hydrophobic peptides, low-molecular weight and water-soluble substances generally cross the intestinal membrane via the paracellular pathway [24]. This is because the transport is mediated mainly by the tight-junction proteins, which form a pore with a diameter of 0.4–0.9 nm in the villi and 5–6 nm in the crypts of the intestinal membrane [25]. In our study, the role of paracellular transport was confirmed with the use of TF3′G, an epithelial barrier enhancer that enhances the expression of adenosine monophosphate (AMP)-activated protein kinase mediated ZO-1, claudin-1, and occludin [26]. The transport of γ-EV was significantly decreased in the presence of TF3′G, which described the route to be paracellular (Figure 5). It has been demonstrated that the paracellular pathway is the most common route of transport for many bioactive peptides, such as VPP [27], VLPVP [28], RVPSL [18], HLPLP [20], and GPRGF [21]. In our study, γ-EV also was shown to be transported through the PepT1 transporter (Figure 6), which is consistent with previous reports suggesting that most di- and tripeptides are transported through PepT1. Many bioactive peptides, such as YPI [18], IPP [29], IQW [19], LSW [16], and IRW [22], have been reported to be transported via both the paracellular and PepT1 routes, while some peptides are transported via both the paracellular and transcytosis routes. However, so far the existing literature evidence does not support the transport of a dietary bioactive peptide via both transcytosis and PepT1, nor any peptide that transports via all three pathways. This is probably related to the lipid membrane or energy dependence, and further research is required to clarify the understanding [5].

Earlier studies have shown that at lower peptide concentrations (≤*K_m_*), the transporter PepT1 is the primary contributor to the total transport rate for the peptides; however, paracellular transport is mostly concentration-dependent. As the concentration increases, the role of paracellular transport becomes more evident. This is because the PepT1 transporter becomes saturated at *V_max_*, and any further increases in the total peptide transport correspond to the passive transport [5]. This could potentially be the reason for the enhanced rate of transport of γ-EV in the initial 2 h. Furthermore, our study also suggested that the expression of tight-junction proteins was not altered at concentrations of 1 and 2.5 mM, but a decrease in occludin expression was observed in the case of 5 mM (Figure S3). The transport of γ-EV had a P_app_ in the range of 1.5–2.5 × 10^−6^ cm/s, depending on the concentration, which was comparable to other bioactive peptides reported in the literature [30]. As previously reported, bioactive peptide transport across the Caco-2 monolayer usually is in the range of 1.0 × 10^−7^ to 10 × 10^−7^ cm/s [5,16,19,24,31,32], and this is similar to any other bioactive food components, such as flavonoids, catechins, and anthocyanins [33,34]. In addition to Caco-2 being a good experimental model in terms of simulating the human intestinal membrane, it has its disadvantages as well. One such disadvantage is the lower expression of uptake transporters as compared to the human intestine, and thus the peptide permeability through Caco-2 may not be an accurate representation of the actual transport through the human intestinal membrane [21,24]. Although the permeabilities of bioactive peptides are generally low (mainly less than 1%) [35], many peptides have still exerted their bioactivities in vivo, after being absorbed in the bloodstream [36,37,38].

## 5. Conclusions

The anti-inflammatory γ-glutamyl dipeptide, γ-EV, was not toxic to the intestinal epithelial Caco-2 cells up to a concentration of 5 mM, and the peptide could be transported intact across the epithelial monolayer without becoming degraded by peptidases present on the apical surface of the intestinal cells. A higher peptide dose of up to 5 mM was found to be safe and enhanced the permeability of the peptide across the intestinal membrane; however, it was shown to modulate the structural tight-junction protein, so future investigation is required to decipher the long-term effect of γ-EV on intestinal tight-junction proteins. Furthermore, the route of transport of γ-EV across the intestinal monolayer was found to be via the PepT1 and paracellular pathways. Future studies are required to explore the in vivo bioavailability of γ-EV; however, the current findings highlight the potential of the application of γ-EV in the formulation of nutraceuticals or functional foods.

## Data Availability

The data presented in this study are openly available in FigShare at https://doi.org/10.6084/m9.figshare.14466414.v1 (accessed on 23 April 2021), https://doi.org/10.6084/m9.figshare.14471238.v1 (accessed on 23 April 2021), and https://doi.org/10.6084/m9.figshare.14466417.v1 (accessed on 23 April 2021).

## Supplementary Materials

The following are available online at https://www.mdpi.com/article/10.3390/nu13051448/s1, Figure S1: Stability of γ-EV during transport through the Caco-2 monolayer cells, Figure S2: Western immunoblotting of the tight junction proteins for the time-dependent transport study of γ-EV, Figure S3: Western immunoblotting of the tight junction proteins for the dose-dependent transport study of γ-EV, Figure S4: Western immunoblotting of the tight junction proteins for the mechanism of transport study of γ-EV.
