# Supplementary material for: Transport of Dietary Anti-Inflammatory Peptide, γ-Glutamyl Valine (γ-EV), across the Intestinal Caco-2 Monolayer
Source: Nutrients. 2021 Apr 24;13(5):1448. doi: 10.3390/nu13051448 (PMC8145144; doi:10.3390/nu13051448)
Supplement: Supplementary file 1 [file nutrients-13-01448-s001.zip › nutrients-1196616-supplementary.pdf]

Supplementary Material

Transport of dietary anti-inflammatory peptide, γ-gluatmyl valine (γ-EV), across the intestinal Caco-2 monolayer

Snigdha Guha[a], Sophie Alvarez[b], Kaustav Majumder[a*]

[a] Department of Food Science and Technology, University of Nebraska-Lincoln, NE, USA

[b] Proteomics and Metabolomics Facility, Nebraska Center for Biotechnology, University of Nebraska-Lincoln, NE, USA

***Corresponding author:**
Dr. Kaustav Majumder, Email: kaustav.majumder@unl.edu

Table of content

1.	**Figure S1.** Stability of γ-EV during transport through the Caco-2 monolayer cells	ii
2.	**Figure S2.** Western immunoblotting of the tight junction proteins for the time-dependent transport study of γ-EV	iii
3.	**Figure S3.** Western immunoblotting of the tight junction proteins for the dose-dependent transport study of γ-EV	iv
4.	**Figure S4.** Western immunoblotting of the tight junction proteins for the mechanism of transport study of γ-EV	v

FIGURES

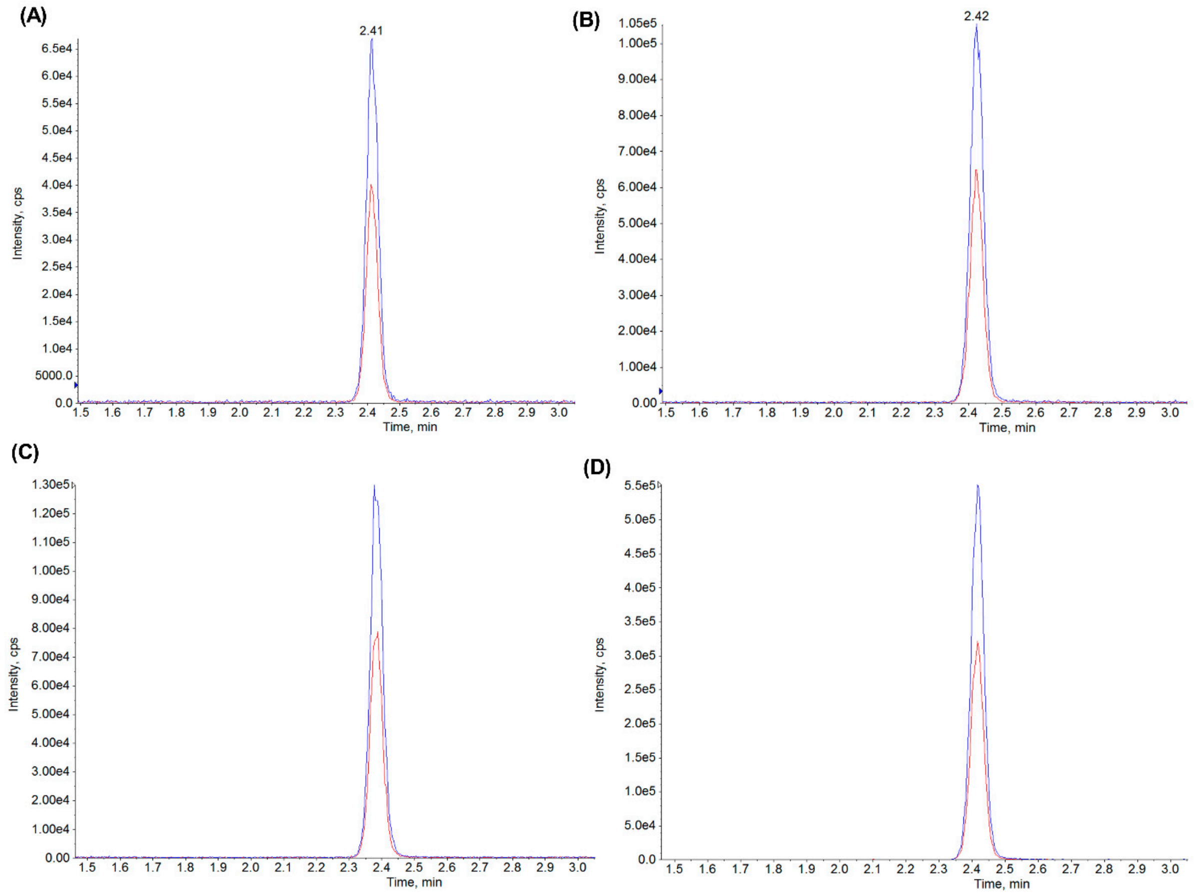

Figure S1: Stability of γ-EV during transport through the Caco-2 monolayer cells. (A) MRM chromatogram of γ-EV in the basolateral solution after 4 h of transport. (B) MRM chromatogram of γ-EV in the basolateral solution after 6 h of transport. (C) MRM chromatogram of γ-EV in the basolateral solution after 2 h of transport for the 2.5 mM dose. (D) MRM chromatogram of γ-EV in the basolateral solution after 2 h of transport for the 5 mM dose.

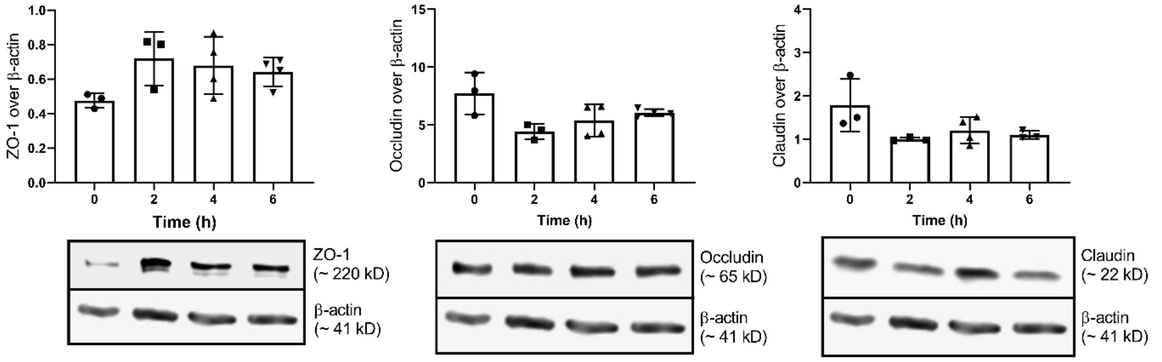

Figure S2: Western immunoblotting of the tight junction proteins for the time-dependent transport study of γ-EV. The expression levels of the three tight junction proteins, (A) ZO-1, (B) Occludin, and (C) Claudin-1, did not differ significantly among the different time periods of 2, 4, and 6 h, during the transport of γ-EV across the Caco-2 monolayer cells. Data presented as the mean ± SD of at least three independent experiments.

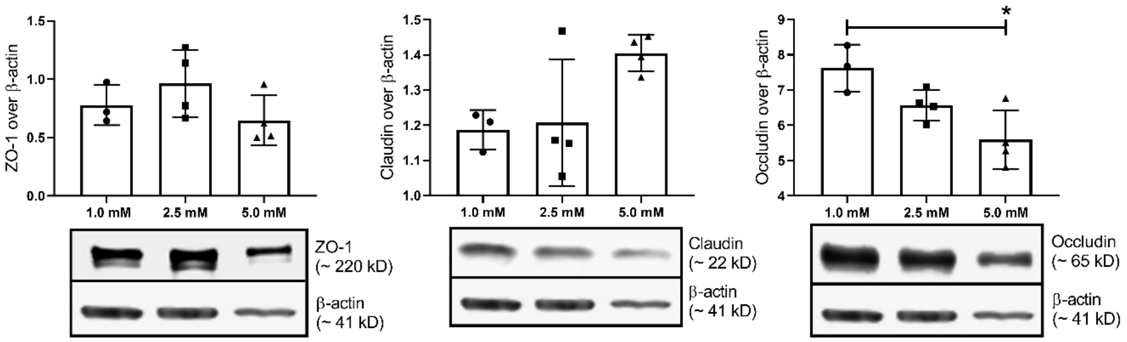

Figure S3: Western immunoblotting of the tight junction proteins for the dose-dependent transport study of γ-EV. The expression levels of the tight junction proteins, (A) ZO-1, and (B) Claudin-1, did not differ significantly among the different doses of 1, 2.5, and 5 mM during the transport of γ-EV across the Caco-2 monolayer cells. However, in case of (C) Occludin, a reduction ($p <0.05$) was seen in the case of 5 mM dose during the transport. Data presented as the mean ± SD of at least three independent experiments. Statistically, the data are represented as $p < 0.001$ for (***), $p < 0.01$ for (**), and $p < 0.05$ for (*).

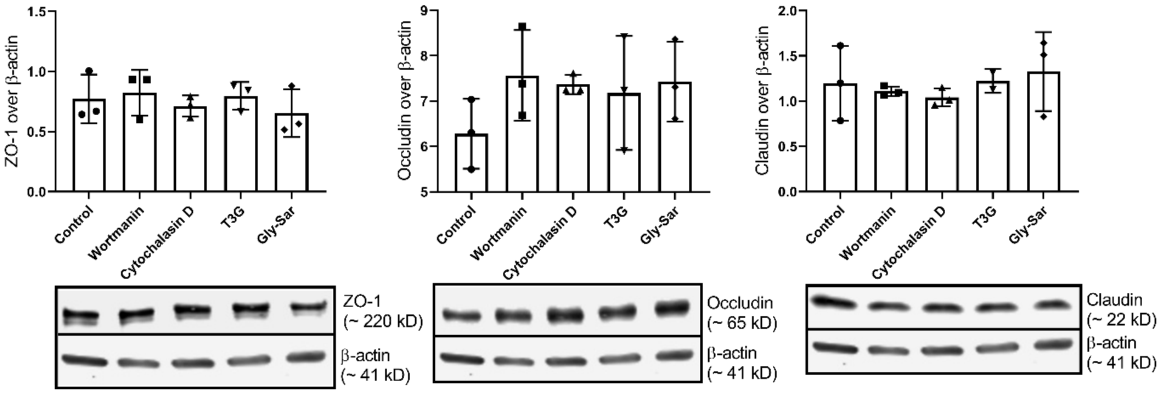

Figure S4: Western immunoblotting of the tight junction proteins for the mechanism of transport study of γ-EV. The expression levels of the three tight junction proteins, (A) ZO-1, (B) Occludin, and (C) Claudin-1, did not differ significantly among the different transport inhibitor treatments (wortmannin, cytochalasin D, TF3'G, and Gly-Sar), during the transport of γ-EV across the Caco-2 monolayer cells. Data presented as the mean ± SD of at least three independent experiments.
